# IBDsite: a Galaxy-interacting, integrative database for supporting inflammatory bowel disease high throughput data analysis

**DOI:** 10.1186/1471-2105-13-S14-S5

**Published:** 2012-09-07

**Authors:** I Merelli, F Viti, L Milanesi

**Affiliations:** 1 Institute for Biomedical Technologies, National Research Council, Via Fratelli Cervi, 93, Segrate (Mi), Italy

## Abstract

**Background:**

Inflammatory bowel diseases (IBD) refer to a group of inflammatory conditions concerning colon and small intestine, which cause socially uncomfortable symptoms and often are associated with an increased risk of colon cancer. IBD are complex disorders, which rely on genetic susceptibility, environmental factors, deregulation of the immune system, and host relationship with commensal microbiota. The complexity of these pathologies makes difficult to clearly understand the mechanisms of their onset. Therefore, the study of IBD must be faced exploiting an integrated and multilevel approach, ranging from genes, transcripts and proteins to pathways altered in affected tissues, and carefully considering their regulatory mechanisms, which may intervene in the pathology onset. It is also crucial to have a knowledge base about the symbiotic bacteria that are hosted in the human gut. To date, much data exist regarding IBD and human commensal bacteria, but this information is sparse in literature and no free resource provides a homogeneously and rationally integrated view of biomolecular data related to these pathologies.

**Methods:**

Human genes altered in IBD have been collected from literature, paying particular interest for the immune system alterations prompted by the interaction with the gut microbiome. This process has been performed manually to assure the reliability of collected data. Heterogeneous metadata from different sources have been automatically formatted and integrated in order to enrich information about these altered genes. A user-friendly web interface has been created for easy access to structured data. Tools such as gene clustering coefficients, all-pairs shortest paths and pathway lengths calculation have been developed to provide data analysis support. Moreover, the implemented resource is compliant to the Galaxy framework, allowing the collected data to be exploited in the context of high throughput bioinformatics analysis.

**Results:**

To fill the lack of a reference resource for 'omics' science analysis in the context of IBD, we developed the IBDsite (available at http://www.itb.cnr.it/ibd), a disease-oriented platform, which collects data related to biomolecular mechanisms involved in the IBD onset. The resource provides a section devoted to human genes identified as altered in IBD, which can be queried at different biomolecular levels and visualised in gene-centred report pages. Furthermore, the system presents information related to the gut microbiota involved in IBD affected patients. The IBDsite is compliant with all Galaxy installations (in particular, it can be accessed from our custom version of Galaxy, http://www.itb.cnr.it/galaxy), in order to facilitate high-throughput data integration and to enable evaluations of the genomic basis of these diseases, complementing the tools embedded in the IBDsite.

**Conclusions:**

Lots of sparse data exist concerning IBD studies, but no on-line resource homogeneously and rationally integrate and collect them. The IBDsite is an attempt to group available information regarding human genes and microbial aspects related to IBD, by means of a multilevel mining tool. Moreover, it constitutes a knowledge base to filter, annotate and understand new experimental data in order to formulate new scientific hypotheses, thanks to the possibility of integrating genomics aspects by employing the Galaxy framework. Discussed use-cases demonstrate that the developed system is useful to infer not trivial knowledge from the existing widespread data or from novel experiments.

## Background

Inflammatory bowel diseases (IBD) are a group of inflammatory conditions concerning colon and small intestine, which cause socially uncomfortable gastrointestinal symptoms. In particular, Crohn's Disease (CD) and Ulcerative Colitis (UC) present the highest incidence in population and are associated with an increased risk for colon cancer [1].

Understanding the causes of the IBD onset is not trivial, since they are complex polygenetic diseases that involve the contributions of genetic susceptibility [2] (collectively, the loci identified to date represent around 15% of the overall variance of potential disease risk, with a dominant contribution provided by three common NOD2 variants), environmental factors (i.e. diet, stress, antibiotics assumption), deregulation of the immune system and its relationship with the commensal microbiota. In particular, the process leading from a *controlled inflammatory condition*, caused by the physiological and irreplaceable presence of microbial flora in the bowel, to a *pathological inflammation *is still widely unknown [3].

Recent advances in mapping the genetic basis of disease susceptibility, coupled with rapid improvements in characterization of the microbiome in healthy and diseased individuals, offer great hope for the development of new IBD treatments. Nevertheless, several key issues need to be better understood. These include, first of all, the determination of the biomolecular mechanisms responsible for the pathology onset. Another important aspect is the capability to distinguish between individuality in IBD aetiology and commonality in pathogenic effector modules, so that therapies can be tailored appropriately to diverse patient subgroups to suitably restore homeostasis. The influence of microbiome-derived molecules on local and systemic immune responses is another area of great scientific interest, that seems to be crucial to determine the way immune system feeds back into shaping the composition of the microbiome [4].

Due to its complexity, the study of IBD must be faced exploiting an integrated and multilevel approach, ranging from genes, transcripts, proteins and pathways altered in affected tissues and trying to consider the regulatory mechanisms intervening in the pathology onset, paying particular attention to the identification of the involved commensal bacteria. The majority of the data available in literature about IBD are not organized in a database structure: some experiments concern genetics, other resources are related to epidemiological aspects, while most data are endoscopical and histological information useful for basic clinical investigations [5,6]. To date, more than 8000 papers about IBD are reported in PubMed [7], around 15000 assays on human and animal models are stored in ArrayExpress [8] and GEO [9], hundreds of NGS experiments can be downloaded from SRA [10] and ENA [11]. Nevertheless, all of them are sparse in literature, and no on-line resources have been developed to homogeneously and rationally integrate biomolecular data related to IBD.

Knowledge-base systems are of critical importance for supporting the formulation and validation of novel hypotheses relying on available genome wide association approaches, target re-sequencing experiments, epigenetics evaluations, RNA-sequencing analysis, proteomics studies, metagenomics or metatranscriptomics experimental designs.

Our approach was to develop the IBDsite, a disease-oriented platform, which collects data related to biomolecular mechanisms altered in IBD. This site records human and bacterial information related to CD and UC, and exploits data integration strategies and analysis tools to help inferring not trivial knowledge from the existing widespread data or for analysing novel experimental data. The IBDsite is freely available at the URL http://www.itb.cnr.it/ibd. The infrastructure is also compliant with the Galaxy platform [12], as it can be experimented using our local instance of the Galaxy (freely available at the URL http://www.itb.cnr.it/galaxy), which presents the IBDsite among the list of data sources, thus promoting analysis on IBD related genomic high-throughput users' data.

The IBDsite aims at representing a first attempt to knowledge and discovery integration of IBD biomolecular aspects by collecting human genes involved in IBD (and providing a hierarchical annotation of the samples) together with data about the involved commensal bacteria. To our knowledge, this represents an important and innovative reference in this field. On the other hand, the presented platform enables the integration with the Galaxy system, thus promoting the analysis of novel genomic experimental data (i.e. sequencing and microarray) in the perspective of enhancing the knowledge about IBD.

## Methods

**Figure 1:**
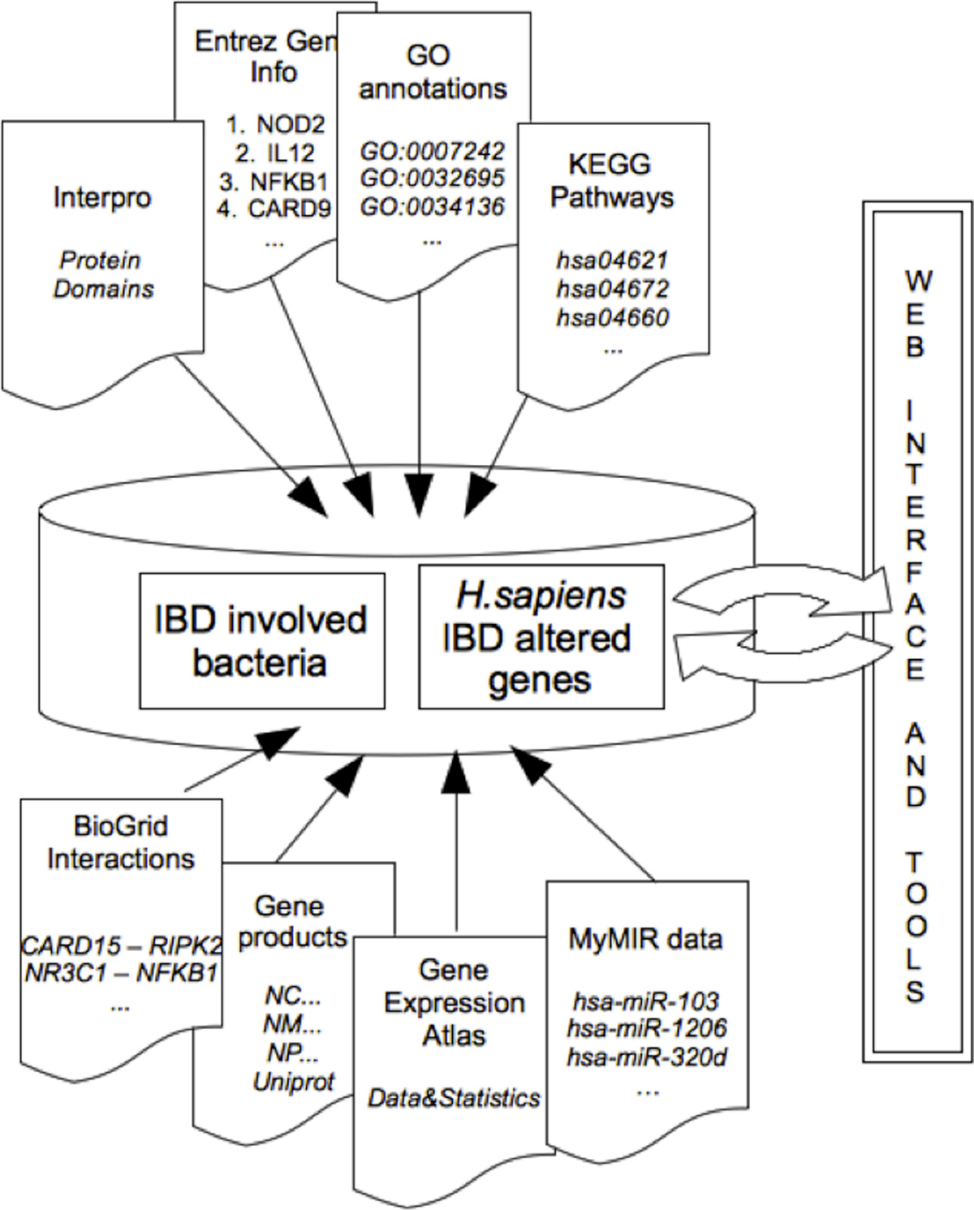
**The IBDsite data schema**. Conceptual schema of the information content maintained in IBDsite. Data about *H. sapiens *genes that correlate to IBD are structured according to a gene-centric view. Annotation tables are related to the main one according to the gene identifiers, also used for structuring the ontology support and the systems biology layer. For commensal bacteria knowledge, the main table maintains data from literature, associating specific bacteria with the pathology and alteration mechanisms.

The IBDsite relies on the MySQL database management system for data maintenance, which enables collecting and formatting heterogeneous data from different sources, in order to make them accessible to the scientific community through a unified query schema by means of a web interface (Figure 1). Information is structured according to a gene-centric view, which means that the central table of the database contains PubMed references associating specific human genes to IBD. Annotation tables are related to the main one according to the gene identifiers, which are also used for structuring the ontology support and the systems biology layer. Even concerning commensal bacteria, the main table maintains data from literature and reports PubMed identifiers of scientific papers associating specific bacteria with the pathology and the related altered mechanisms. Whenever possible, a direct correlation is made between human genes and bacterial species causing the dysbiosis. A table dedicated to taxonomy information (retrieved from the NCBI Taxonomy [13]) has been populated for a better characterization of disease-involved bacteria.

The primary data collection about genes involved in IBD has been performed manually from literature, which in the authors' opinion is an added value of the implemented database, since it guarantees a high reliability of the resource. This manual process will be performed periodically, according to novel papers published in this field.

Annotation data have been retrieved from diverse resources, differently formatted and organized, that includes: Gene Ontology [14] for stardardized gene products annotation terms; RefSeq [15] for sequence data; Entrez Gene [16] for gene annotation; Uniprot [17] and Protein Data Bank (PDB) [18] for protein annotation and structure; InterPro [19] for what concerns protein domains; Biogrid [20] for proteins interections; KEGG [21] and Reactome [22] for pathways data. Gene Expression Atlas [8] has been queried for retrieving transcription profiles while MyMir [23] for retrieving predictions about miRNA-targets relation. The database considered as primary source for sequences concerning metagenomics and metatranscriptomics experiments context is the NCBI Genome/Bacteria database [24].

Different scripts for downloading annotation data regarding genes involved in IBD were implemented ad hoc for each resource, in a typical data warehouse fashion. Repositories that enable batch download modes, such as NCBI maintained databases, have been queried following standardized approaches (i.e. web services), while download has been enabled through suitable *http *links or *ftp *sections for resources without direct access to data. In both cases, the developed system is robust even in the case of changes to the original resources, since data are typically provided in structured formats. Retrieved information was parsed and formatted suitably to populate the database tables: this enables an easy data access (through sql query language) and the creation of a user-friendly interface. Monthly maintenance will be performed by the system developers to keep updated the IBD repository data. Users will be acknowledged of the latest updates.

In order to exploit the IBDsite knowledge base for the analysis of biomolecular high-throughput experiments, the system was designed to be a source of data for the Galaxy platform, one of the most popular frameworks for genomic sequences management, their alignments, and functional annotation. This integration can be tested in our local instance of this framework, called Galaxy@ITB, through which all the examples in the following section were performed. The IBDsite was inserted among the data sources of Galaxy@ITB, by defining an XML configuration file, which instructs the system in loading the file provided by the external source. This configuration allows the user to select a set of genes of interest from the IBD database and to exploit it as knowledge base for analysing external datasets such as novel experimental results or literature datasets from recognised repositories.

## Results

### The IBDsite

**Figure 2:**
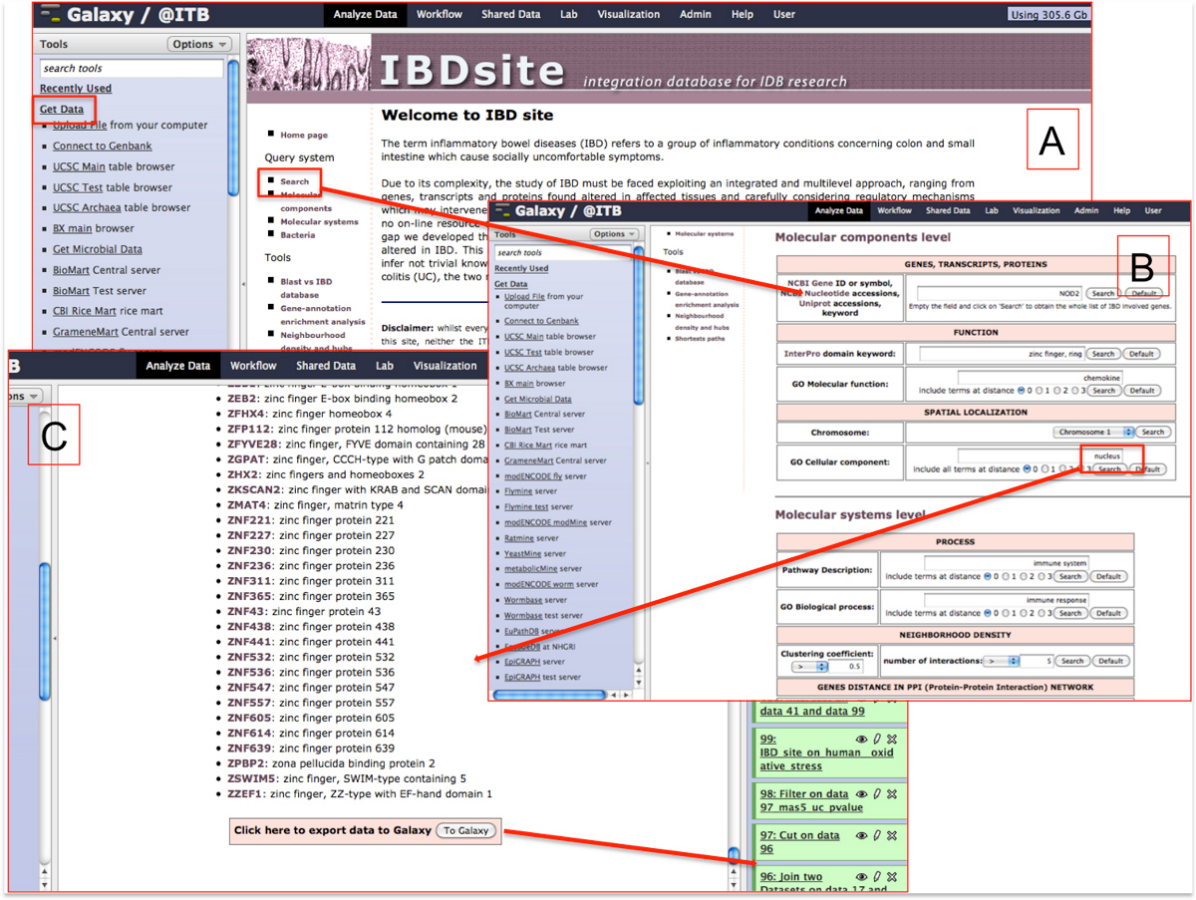
**IBDsite features**. Examples of features of the IBDsite integrated with the Galaxy@ITB framework. A) Through the IBDsite link in the 'Get Data' list the maintained data are accessible in the Galaxy platform. B) The menu on the left of the site allows to browse the information (the Search button provides query masks). C) The retrieved list of data can be sent to the Galaxy framework and appear, as usual, on the right side, where data can be chosen to perform desired analysis.

The web interface of the IBDsite (Figure 2) has been implemented in PHP and JavaScript languages and provides tools to query, show and analyse data collected in the resource. Queries on human data can be performed through gene or protein identifiers, synonyms and descriptions, or information about gene products function, represented by the protein domains and the GO Molecular Functions. Moreover, it is possible to query the system considering the genomic region (spatial coordinates mapped on the UCSC Genome browser [25]) or the cell compartment, a feature accomplished by using the GO Cellular Components annotation. Finally, the IBDsite can be queried from a specific biochemical pathway name, exploiting KEGG, Reactome and GO Biological Processes data: in this context, the user interested in a specific biological process can retrieve the list of genes found involved in IBD.

For each human gene known to be involved in IBD a report is available. IBD genes are annotated by gene symbol, description, aliases, sequences and related molecular alterations with reference to the papers where are described. Collected evidence concerns genomic variations such as Single Nucleotide Polymorphisms (SNPs) occurring in genes and their flanking regions, gene expression level, and transcription and translation regulation factors. Gene expression information is supported by a link towards the Gene Expression Atlas [8] report page, where the over/under expression of the specific gene in CD and UC is listed. Each gene is also associated with microRNAs that are predicted to target the gene transcripts, as retrieved from myMIR site. Gene products have been collected as lists of mRNA sequences and related protein isoforms according to the NCBI RefSeq. They are annotated with all the identifiers suitable to download the related sequences, with functional domains according to InterPro and structural models from the PDB. Furthermore, the IBDsite retrieves information regarding biochemical networks where the gene is involved as reported in KEGG and Reactome and the protein-protein interactions (PPIs) annotated in BioGRID.

The immunological aspect of the IBD onset must be evaluated in the perspective of patients' microbiome composition. Therefore, it is crucial to consider the presence (and the quantity) of commensal bacteria, which are known to deregulate the immune system in UC and CD. A whole section of the IBDsite is dedicated to the microbiome members involved in these pathologies. Data about bacteria can be accessed through the 'Bacteria' link in the left menu, where user can query the IBD involved bacteria and the human genes altered in disbiosys. The bacteria report lists the bacterial taxonomy information together with the reference papers about their involvement in the considered pathologies, and the correlated altered human genes, if known: in the latter case both these data are also visualized on the genes report page, thus providing a complete overview of the host/commensal relationship.

### Galaxy interaction

IBDsite data can be accessed through the Galaxy platform, in particular, considering the Galaxy@ITB instance of this framework, by clicking on the suitable link listed under *GetData *section (left menu). The interaction between the IBDsite and Galaxy allows to import information that enable the analysis of novel data in the light of the IBDsite knowledge content. Genomics data can be uploaded in the system either from the local file system or by downloading them directly from standardized remote repositories of high-throughput biomolecular data, such as SRA for short read sequences, or GEO and ArrayExpress for gene expression microarrays. This is possible in Galaxy@ITB by employing customized Galaxy tools (i.e. the *genebank *plugin and the *Rgenetics *package).

This integration strategy is oriented to *H. sapiens *genes and gut bacteria genomics data, with particular concern to next generation sequencing data, both for DNA and RNA sequences, genotyping data, and gene expression microarrays.

The procedure for sequences analysis consists in different pipelines of semi-standardized steps, provided by the Galaxy framework and customizable for specific requirements. Generally, these steps include data quality evaluation, sequence trimming, data mapping on human and bacteria genomes, differential expression evaluation, and phylogenetic analysis. Relying on the features enabled by Galaxy@ITB, reads can be visualized on many different genome browsers (UCSC Genome Browser [25], Ensembl [26], GBrowse [27], GeneTrack [28], Trackster [29]), thus allowing the evaluation of genetic alterations (in case of DNA sequences), or modulations in gene expression (in case of RNA alignments). Both IBDsite data and Galaxy@ITB information refer to the hg19 (GRCh37) release of the human genome, thus being comparable and displayable on the same genome track. Therefore, the whole set of IBD genes maintained in the IBDsite can be visualized with experimental data in the Galaxy@ITB framework, supporting the analysis of the alterations found in inflammatory bowel pathologies. Alternatively, IBDsite data can be filtered before mapping (i.e. on the basis of a specific biochemical pathway, or a cellular localization or even a chromosomal position) thus providing a diverse perspective of the analysed data.

## Discussion

In this section we will describe the use of the IBDsite in the context of some typical Galaxy based analysis. The system, in the version integrated into Galaxy@ITB, has been used to perform analysis on publicly available literature data of gene expression microarray, metagenomics experiments and RNA-sequences.

### Genome wide analysis microarray

We exploited data produced in a genome-wide expression study on 36 human samples, performed at the John Hopkins School of Medicine [30]. Here single endoscopic pinch biopsies were used to elucidate patterns of gene expression in active and inactive areas of UC and CD, even considering indeterminate colitis (IC) and infectious colitis (IF), in order to identify pathogenic processes underlying these disease subtypes. Experiments have been performed using Affymetrix 'Human Genome U95 Version 2' chips. Unsupervised classification of different biopsies suggested that diverse sample types present distinctive expression patterns. The most important results concern the identification of differentially expressed genes (DEGs) in CD vs. normal samples (mainly IFITM1, IFITM3, STAT1, STAT3, TAP1, PSME2, and PSMB8), and in UC vs. normal conditions (mainly HNF4G, KLF5, AQP8, ATP2B1, and SLC16A).

By employing a customized version of the *Rgenetics *plugin of Galaxy [31], microarray raw data have been retrieved from the GEO repository (identifier: GSE6731) and processed with different R-Bioconductor tools integrated within our Galaxy@ITB framework, in order to define the differentially expressed genes. Data have been normalized with two main methods, MAS5 [32] and RMA [33], calculating both the *p-value *and the *fold change *of the microarray results. Parameters employed for these analyses (respectively: *p-value *< 0.002 and absolute value of *fold-change *> 2) have been defined in order to point out real differences in the gene expression of affected and unaffected patients. Although the original analysis was performed with a different pipeline (relying on DChip [34] and SAM [35]), results present a considerable overlap both for CD and UC.

The added value provided by the IBDsite integration to these analyses is the possibility to reconsider data in the view of our knowledge base, by evaluating for example only specific pathways or biological processes involved in IBD. The whole perspective of the analysis can be reverted, by mapping expression of genes belonging to specific networks (working also below the given threshold), instead of inferring annotation only of differentially expressed genes (like it is common to proceed).

Concerning biomolecular pathways and biological processes, the IBDsite can show genes involved in specific networks related to IBD. Dataset transmitted to Galaxy@ITB reports genes included into specific pathways: those altered in IBD are labelled and can be visualized as separate subsets. For example, we intersected the DEGs related to microarray data, achieved using fold change values calculated with the MAS5 algorithm, with genes of different pathways. Results are reported in Table 1. The table shows that the correlation of the IBD onset with the *immune system pathway*, although largely expected, comes out clearly when genes of this pathway are used to filter DEGs.

**Table 1:** Functional annotation of expression microarray.

	Immune System	Cytokine interaction	Inflammatory response	Apoptosis (GO term)
UC (297)	22	14	12	4
CD (77)	15	16	12	2
IF (353)	16	10	14	4
IC (1226)	30	20	13	15

Number of Differential Expressed Genes (DEGs) involved in diverse pathways for different IBD (UC: Ulcerative Disease, CD: Crohn's Disease, IF: infectious colitis, IC: Indeterminate colitis). The total number of DEGs is reported in brackets.

**Figure 3:**
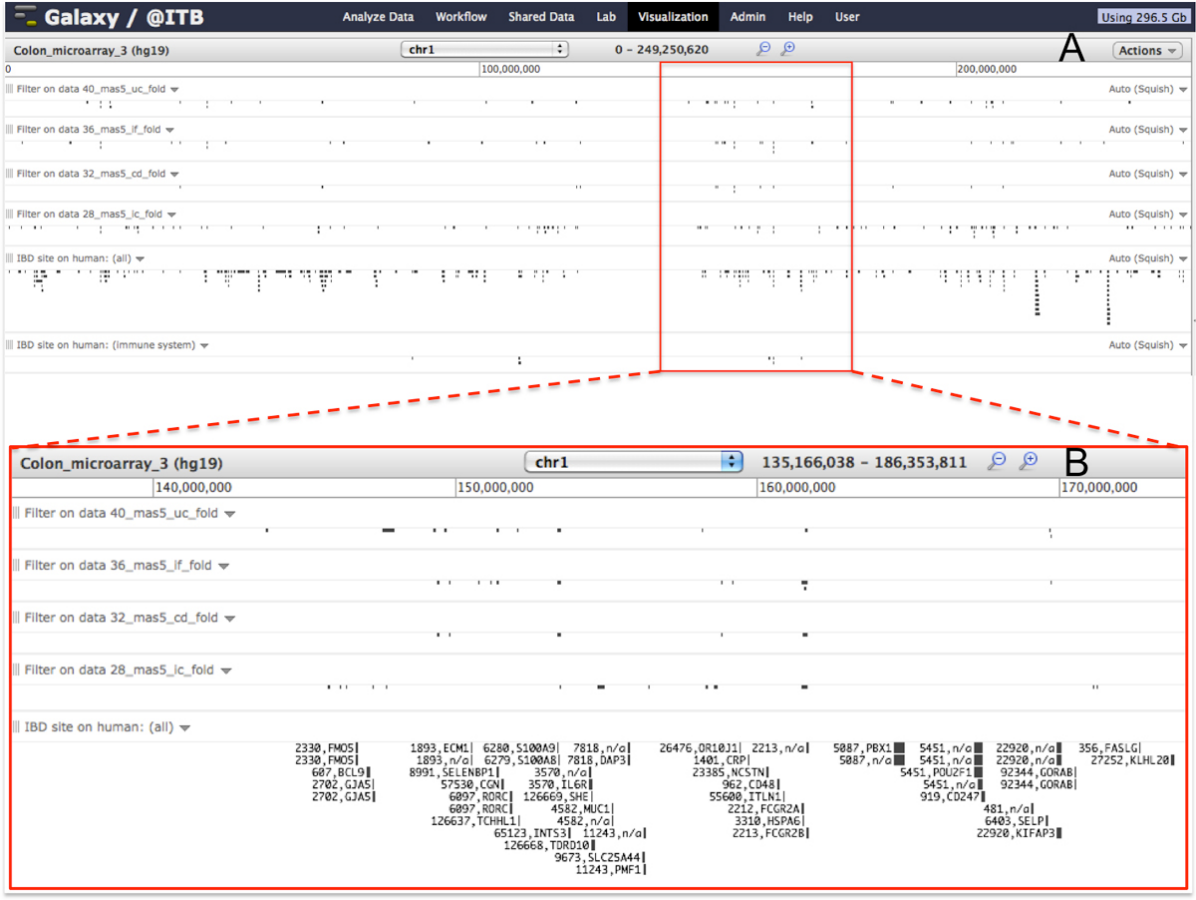
**Examples of differential expression genes in microarray data on Galaxy**. A) Colon microarray data mapped on Chromosome 1. B) Focus on the Chromosome 1 region 135,166,038-186,353,811 that appears to be rich of differentially expressed genes corresponding with genes involved in IBD.

Data can be visualized in one of the genomic browsers available at Galaxy@ITB together with data extracted from the IBDsite, considering, for example, the whole set of genes maintained in the database and the subset related to the immune system. An example of data visualization is reported in Figure 3A, related to human chromosome 1 (also accessible at http://www.itb.cnr.it/sup/ibd/v/colon-microarray).

The immediate and complete vision provided by this integrative visualization on the whole genome allows a clear overview of the involvement of genomic regions in the pathology onset. For example, the chromosome 1 appears as one of the most involved in the four considered diseases (UC, CD, IC, IF), as it can be inferred by the correlation among DEGs found in the four pathology subgroups and the knowledge retrieved from the IBDsite. The possibility to visually integrate this information permits, for example, to recognise region chr1:140,000,000-170,000,000 (Figure 3B), as crucial for the IBD onset. Actually, this chromosomal localization includes many genes known as involved in these pathologies, such as MUC1 [36], CD48 [37], FASLG [38], ECM1 [39] and IL6R [40].

The full set of data and analysis steps used for this use-case are accessible at Galaxy@ITB in the Published History under the name 'Colon Microarray' (available at url: http://www.itb.cnr.it/sup/ibd/h/colon-microarray).

### Metagenomics of the microbiome

The second application example has been performed on metagenomics sequencing data, downloaded from the SRA archive. The considered experiment (identifier: SRP002427) describes the gut microbiome of different patients affected by UC, coming from gut biopsies collected in the frame of the multi-center Ulcerative Colitis Human Microbiome Project (UCHMP), which aims at examining the role of the enteric microbiome in causing human ulcerative colitis.

Starting from the hypothesis that changes in microbiota will precede the onset of IBD, the main target of the study is to determine the role of the gut bacteria in the development of human inflammatory bowel disease using the whole metagenome sequencing technique. The microbial profile of the inflamed area will lead the choice of the most effective antibiotic therapy. Human tissue components have been removed from the samples, thus the aligned sequences should refer only to the bacterial components of the bowel. Data have been locally downloaded and analysed using a Galaxy workflow, which mainly relies on the annotation of sequences using Megablast [41] against the Whole Genome Shotgun sequence assemblies (WGS) database [42]. The annotation results are then analysed through a set of parsers for the identification of the sequences taxonomy, relying on the data fetched from the Taxonomy database available at NCBI, and the creation of a phylogenetic tree.

**Figure 4:**
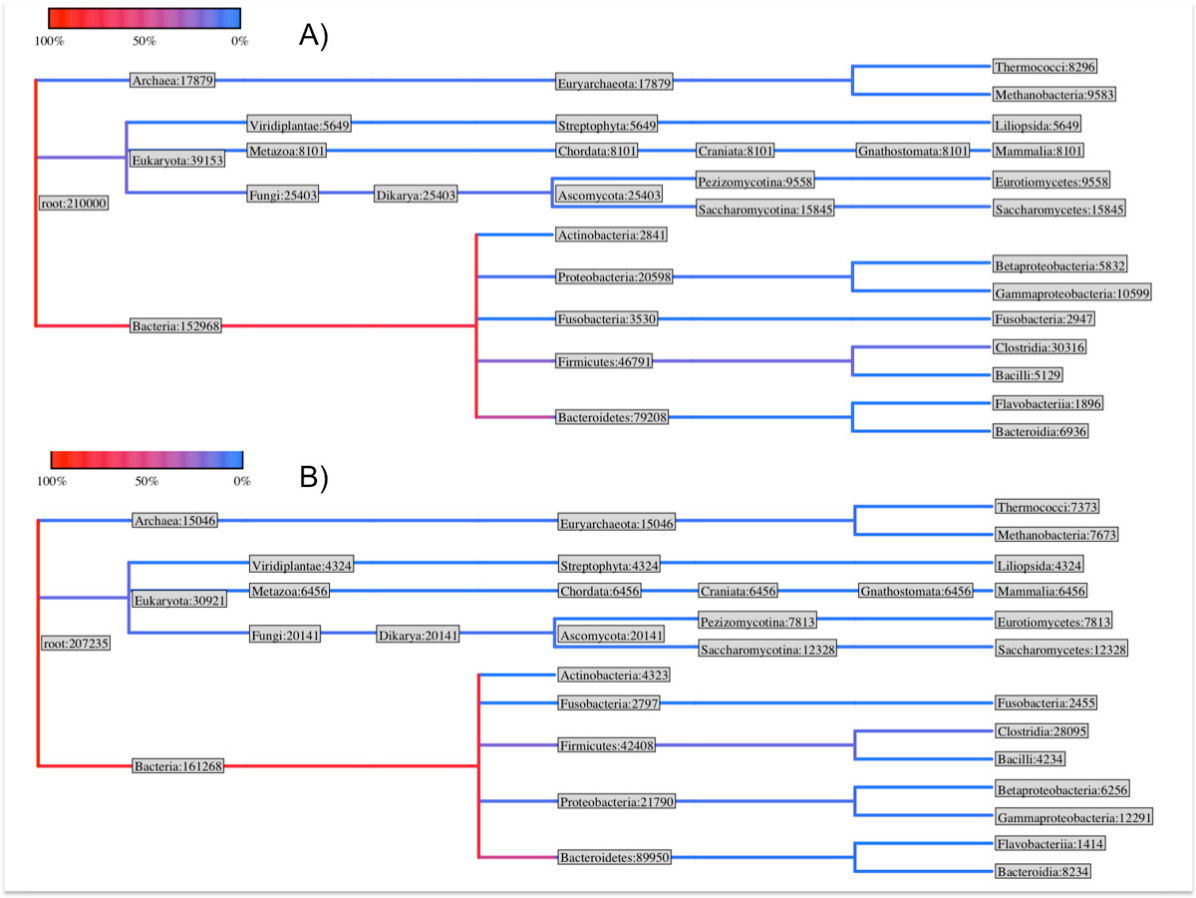
**Phylogenetic distribution**. Phylogenetic distributions of sequences found in the analysed samples of UC affected patient. Colours are related to the cardinality of the datasets.

Using Galaxy@ITB, two samples of commensal bacteria from UC patients (identifier: SRR059128 and SRR059129) have been analysed using the metagenomics workflow, and results about the obtained phylogenetic tree are shown in Figure 4. Moreover, in order to compare the number of reads falling in each particular taxon for the two samples, a Poisson process has been performed, in order to calculate two Z scores (Z1 [43] and Z2 [44]). Results are reported in Table 2.

**Table 2:** Taxonomy distribution over samples

Phylum	Readcount1	Readcount2	Z1	Z2
root	207235	210000	4.281	4.281
Archaea	15046	17879	15.613	15.627
Bacteria	161268	152968	14.806	14.808
Eukaryota	30921	39153	31.098	31.152
Fungi	20141	25403	24.657	24.698
Metazoa	6456	8101	13.634	13.656
Viridiplantae	4324	5649	13.268	13.297
Dikarya	20141	25403	24.657	24.698
Actinobacteria	4323	2841	17.509	17.605
Ascomycota	20141	25403	24.657	24.698
Bacteroidetes	89950	79208	26.118	26.131
Chordata	6456	8101	13.634	13.656
Euryarchaeota	15046	17879	15.613	15.627
Firmicutes	42408	46791	14.675	14.680
Fusobacteria	2797	3530	9.215	9.231
Fusobacteria	2797	2947	1.979	1.979
Proteobacteria	21790	20598	5.790	5.790
Streptophyta	4324	5649	13.268	13.297
Craniata	6456	8101	13.634	13.656
Pezizomycotina	7813	9558	13.240	13.257
Saccharomycotina	12328	15845	20.953	20.995
Gnathostomata	6456	8101	13.634	13.656
Bacilli	4234	5129	9.249	9.260
Bacteroidia	8234	6936	10.539	10.548
Betaproteobacteria	6256	5832	3.856	3.857
Clostridia	28095	30316	9.190	9.191
Eurotiomycetes	7813	9558	13.240	13.257
Flavobacteriia	1414	1896	8.378	8.400
Fusobacteria	2455	3530	13.896	13.952
Fusobacteria	2455	2947	6.694	6.701
Gammaproteobacteria	12291	10599	11.183	11.191
Liliopsida	4324	5649	13.268	13.297
Mammalia	6456	8101	13.634	13.656
Methanobacteria	7673	9583	14.540	14.562
Saccharomycetes	12328	15845	20.953	20.995
Thermococci	7373	8296	7.374	7.377
Eurotiomycetidae	7813	9558	13.240	13.257
Commelinids	4324	5649	13.268	13.297
Euarchontoglires	6456	8101	13.634	13.656
Bacteroidales	8234	6936	10.539	10.548
Clostridiales	28095	30316	9.190	9.191
Enterobacteriales	12291	10599	11.183	11.191
Eurotiales	7813	9558	13.240	13.257
Flavobacteriales	1414	1896	8.378	8.400
Fusobacteriales	2455	2947	6.694	6.701
Lactobacillales	4234	5129	9.249	9.260
Methanobacteriales	7673	9583	14.540	14.562
Neisseriales	6256	5832	3.856	3.857
Poales	4324	5649	13.268	13.297
Primates	6456	8101	13.634	13.656
Saccharomycetales	12328	15845	20.953	20.995
Thermococcales	7373	8296	7.374	7.377
Haplorrhini	6456	8101	13.634	13.656
Hominoidea	6456	8101	13.634	13.656
Bacteroidaceae	8234	6936	10.539	10.548
Clostridiaceae	2545	3048	6.726	6.733
Enterobacteriaceae	12291	10599	11.183	11.191
Fusobacteriaceae	2455	2947	6.694	6.701
Hominidae	6456	8101	13.634	13.656
Lachnospiraceae	7272	9245	15.352	15.379
Methanobacteriaceae	7673	9583	14.540	14.562
Neisseriaceae	6256	5832	3.856	3.857
Poaceae	4324	5649	13.268	13.297
Ruminococcaceae	18278	18023	1.338	1.338
Saccharomycetaceae	12328	15845	20.953	20.995
Streptococcaceae	4234	5129	9.249	9.260
Thermococcaceae	7373	8296	7.374	7.377
Trichocomaceae	7813	9558	13.240	13.257
Homininae	6456	8101	13.634	13.656
Pooideae	4324	5649	13.268	13.297
Triticeae	4324	5649	13.268	13.297
Aegilops	4324	5649	13.268	13.297
Bacteroides	8234	6936	10.539	10.548
Buchnera	6526	4039	24.196	24.368
Clostridium	2545	3048	6.726	6.733
Emericella	7813	9558	13.240	13.257
Escherichia	3423	4023	6.953	6.959
Faecalibacterium	8768	7754	7.889	7.892
Fusobacterium	2455	2947	6.694	6.701
Homo	6456	8101	13.634	13.656
Kluyveromyces	6161	7431	10.893	10.905
Methanothermobacter	7673	9583	14.540	14.562
Neisseria	6256	5832	3.856	3.857
Pyrococcus	7373	8296	7.374	7.377
Ruminococcus	9510	10269	5.397	5.398
Saccharomyces	6167	8414	18.608	18.664
Aegilops tauschii	4324	5649	13.268	13.297
Bacteroides thetaiotaomicron	8234	6936	10.539	10.548
Buchnera aphidicola	6526	4039	24.196	24.368
Emericella nidulans	7813	9558	13.240	13.257
Escherichia coli	3423	4023	6.953	6.959
Faecalibacterium prausnitzii	8768	7754	7.889	7.892
Fusobacterium nucleatum	2455	2947	6.694	6.701
Homo sapiens	6456	8101	13.634	13.656
Kluyveromyces lactis	6161	7431	10.893	10.905
Methanothermobacter thermautotrophicus	7673	9583	14.540	14.562
Neisseria gonorrhoeae	6256	5832	3.856	3.857
Pyrococcus horikoshii	7373	8296	7.374	7.377
Ruminococcus gnavus	5255	6709	13.293	13.318
Ruminococcus torques	4255	3560	7.862	7.870
Saccharomyces cerevisiae	6167	8414	18.608	18.664

List of the Z-scores obtained using Poisson distribution on the analysed metagenomics data. This statistical evaluation highlights the correlation between the reads distribution in the taxonomy found in the samples from patient with UC and CD.

Using the information available in the IBDsite, it is possible to annotate the phylogenetic tree computed by Galaxy@ITB in order to correlate information regarding the identified microbioma alterations and the gene correlated to this disbiosys. In particular, for each leaf of the phylogenetic dendrogram it is possible, by means of the IBD site knowledge base, to correlate the pathologies (and the PubMed identifier of the literature reference), the experiment type, the sample type, the involved human genes and additional notes when available. In the example, this annotation helps in correlating the disbiosys with mucosal cytokine and chemokine profiles, in particular for interleukin-1 beta (IL-1 beta), interleukin-6 (IL-6), interleukin-8 (IL-8) and TNF alpha [45-47].

Data and analysis steps used for this analysis are accessible at the Galaxy@ITB site in the Published History under the name 'Colon Metagenomic', through the link http://www.itb.cnr.it/sup/ibd/h/colon-metagenomics.

### Analysis of colon RNA sequencing

A last example is within the context of human RNA sequencing. In this case we chose to consider samples of patients affected by colon cancer versus normal samples, since no data are available concerning RNA-sequencing in relation to IBD affected patients. Nevertheless, the choice is well fitted with the research on IBD, since colon cancer onset is the most diffuse follow-up of inflammatory bowel diseases. RNA-sequencing data have been automatically downloaded from the SRA repository at NCBI using the *genebank *plugin, available at our local instance of Galaxy.

In detail, four runs have been downloaded for this example (normal sample identifiers: SRX066252 and SRX066254; tumour sample identifiers: SRX066253 and SRX066255), which describe the expression profile by high throughput sequencing (Illumina Genome Analyzer) of human colon cancer and normal samples of two affected patients. Data have been processed using a well-established workflow for next generation sequencing analysis, which mainly relies on the bowtie algorithm [48], in order to check the quality, filter and align reads against the reference genome. The pipeline steps are accessible in the Galaxy@ITB site in the Published History under the 'Colon cancer RNA-seq' identifier (http://www.itb.cnr.it/sup/ibd/h/colon-cancer).

Mapped results, both tumour affected and normally affected ones, have been visualized in the same track, together with the IBDsite data, to present the intersections among the differentially expressed genes and the set of genes known to be involved in inflammatory bowel disorders. Data visualization is available at the following link http://www.itb.cnr.it/sup/ibd/v/colon-cancer.

**Figure 5:**
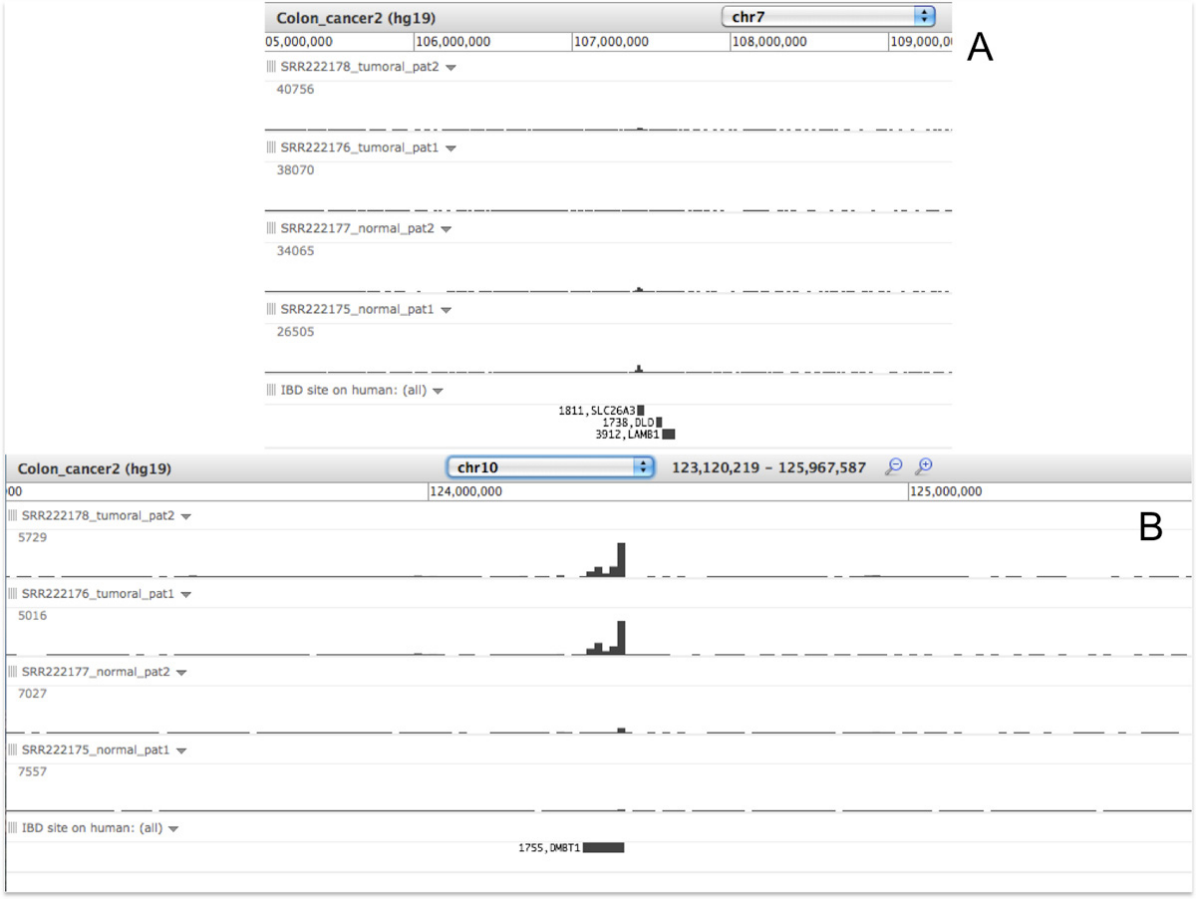
**Examples of differential expression genes in sequencing data in Galaxy**. A) Detail of a genomic region on Chromosome 7, where a differential gene expression emerges between normal and pathological samples sequencing data. This area matches with the SLC26A3 gene, which is known to be involved in IBD. B) Detail of a genomic region on Chromosome 10, where differential gene expression is shown between normal and pathological samples sequencing data. This area matches with the DMBT1 gene, which is known to be involved in IBD.

Some biologically considerations can be sketched immediately from these visualized data. An overview of the whole chromosomes set allows to identify a region with differential expression among normal and colorectal cancer affected patients: this corresponds to the SLC26A3 gene, known to be involved in gastrointestinal pathologies such as congenital chloride diarrhea [49] and ulcerative colitis [50] (Figure 5A). A similar correlation emerges for DMBT1, which is evidently involved in ulcerative colitis [51] and in various cancer types (prostate cancer, breast cancer, glioblastoma, medulloblastoma and melanoma) and that here appears to be differentially expressed even among colon cancer and normal tissues (Figure 5B). Similar considerations can be done for C15orf48 (chr15: 45722763-45725647), which results to be differently expressed in cases and controls and is even known to be involved in gastrointestinal disorders [52].

The inflammatory microenvironment related to the IBD has a prominent role in the onset and progression of colon cancer, but also a plethora of growth factors and cytokines are involved in this process. For example, tumour-necrosis factor-α [53], interleukin-6 [54] and TGF-β [55] seem to be crucial for tumour initiation, because they stimulate the inhibition of the NF-κB pathway in epithelial cells. Similarly, data show an increased expression of the Toll-like receptors, which stimulated by the gut microflora can directly enhance tumorigenesis [56].

### Combining different 'omics' data

Combining different 'omics' data, although highly desirable, results a difficult task to accomplish, because of diverse experimental conditions and analysis inhomogeneity, which often lead to unmatchable data. An attempt to overcome this limitation is the use of a bottom-up approach to data, such as that proposed in the IBDsite. In the IBDsite the layer of manually collected genes allows to browse and integrate different types of data: this strategy is made possible by the interaction with the Galaxy framework, as shown in the previous discussion. By means of the IBDsite genes, information provided by IBD microarrays data with RNA-sequencing data of colon cancer can be combined, to formulate hypotheses about mechanisms underpinning the pathological inflammatory correlation of these diseases with colon cancer. Although RNA-sequencing data in patients affected by IBD would provide more consistency to the description of this process, the analysis performed shows a higher correlation of Ulcerative Colitis genomic patterns with colon cancer in comparison to Crohn's Disease. This aspect clearly emerges in Chromosome 6, Chromosome 10, Chromosome 11, Chromosome 12, Chromosome 16, where microarray derived expression profiles of IC and UC samples resembles the trend of sequencing data in patients with colon cancer much more then CD samples (http://www.itb.cnr.it/sup/ibd/v/micro-cancer). This hypothesis is confirmed by epidemiologic data that describe a higher risk to develop colon cancer in patients with UC with respect to CD [57].

## Conclusions

Inflammatory bowel diseases represent a diffuse and disabling class of pathologies, affecting people that approach their life in a reasonably normal way. Regardless the high incidence and a variety of epidemiologic and genetic studies, causes of the onset and development of IBD remain unclear. The sparse and unstructured knowledge developed in this field neither correctly support the advances in the development of a pathology onset model, nor the identification of effective and personalised therapies for these complex pathologies.

The main targets in the field are the comprehension of the mechanisms underlying the pathology development, the identification of the bacteria involved in the interactions with the human genome, and the identification of the biomolecular hubs where it is possible to intervene with therapies, especially considering the great interindividual variations that can modify the enteric microbial communities.

The presented work tries to fill the lack of a curated IBD knowledge base. The IBDsite represents both an infrastructure to analyse high-throughput experimental data and an integrated collection of homogeneous knowledge, that remains crucial for evaluating new information and to formulate novel scientific hypotheses. The IBDsite maintains, in a structured format, the available biomolecular knowledge about the human genes altered in IBD and the bacteria found involved in their onset, thus acting as a non redundant, consistent and homogeneous container for CD and UC known biomolecular information.

The IBDsite, having the ability of working in connection with the Galaxy platform, provides a crucial possibility of handling new experimental data and exploiting the collected knowledge base information, in order to analyse new samples in the light of the present understanding for discerning the multifactorial mechanisms of the IBD onset.

The proposed infrastructure is capable of handling different types of IBD data and experiments, providing them the appropriate homogeneity in order to be integrated. The possibility of exploiting data from the IBDsite in order to correlate different experiments is an added value to the work: as in all complex pathology, the collection of data at all 'omics' levels is the only affordable way to achieve a complete vision on the involved biomolecular mechanisms.

## Competing interests

The authors declare that they have no competing interests.

## Authors' contributions

IM participated in the design of the study, performed literature analysis and developed the Galaxy interaction of the system. FV participated in the design of the study, performed literature analysis and developed the reference database, developed the web interface and drafted the manuscript. LM coordinated the project and provided access to the computational facilities for maintaining the bioinformatics resources. All authors read and approved the final manuscript.
